# Suggestions for the Busy Doctor

**Published:** 1892-08

**Authors:** 


### Suggestions for the Busy Doctor.

---

**Acute Orchitis.**—In the early stages of acute orchitis give from three to five drops of the tincture of pulsatilla every two hours.—*Exchange.*

---

**To Remove Dandruff.**—The following is given in Lillard's Practical Hints and Formulæ:

| | | |
|---|---|---|
| ℞ | Chloral hydrate | ʒij |
| | Glycerine | ℥j |
| | Bay rum | q. s. oj |

M. —*Exchange.*

---

**Ingrowing Toe-Nail.**—

| | | |
|---|---|---|
| ℞ | Muriatic acid, | |
| | Nitric acid | āā ʒj |
| | Chloride zinc | ℥j |

M. Sig. Apply one drop to the affected part once a day. This gives instant relief to the pain caused by ingrowing toe-nail.

[Operation is the only cure; wide-toed shoes the best preventive.—Ed.]

---

**Disinfectant Mouth Wash.**—Dr. Thomas finds the following a pleasant and efficient buccal disinfectant:

| | | |
|---|---|---|
| ℞ | Thymol | gr. iij |
| | Benzoic acid | gr. xl |
| | Tinc. of eucalyptus | ʒiij |
| | Essence of peppermint | ♏x |
| | Alcohol | ʒiij |

M. d.—Sig. Pour enough into a glass of water to render it turbid, and use as a mouth wash.—*Medical Fortnightly.*

---

**In the Treatment of Affections of the Stomach.**—

| | | | |
|---|---|---|---|
| (a) | ℞ | Phenate of cocaine | 5 centigrammes. |
| | | Subnitrate of bismuth | 2 grammes. |

Mix and divide into five cachets. Sig. In cases of gastralgia, one cachet in the morning, before breakfast, or one hour before the expected principal crisis.

| | | | |
|---|---|---|---|
| (b) | ℞ | Phenate of cocaine | 7 centigrammes. |
| | | Powder of condurango | 1 gramme. |

Mix and divide into ten small cachets. Sig. In cancer of the pylorus, one cachet in the morning before breakfast.—*Univ. Med. Mag.*

**Treatment of Acute and Chronic Nasal Catarrh.**—

℞ Phenate of cocaine . . . . . . . . 20 centigrammes.
Pulverized boric acid . . . . . . . 2 g.ammes.

M. Sig. External use.

---

**In Uterine Hemorrhage.**—Huchard (*Revista de Ciencias Médicas de Barcelona*, April 25, 1892) uses this prescription:

℞ Ergotine,
Sulphate of quinine . . . . . āā 2.00 grammes.
Powder of digitalis,
Extract of hyoscyamus . . . . āā 0.20 gramme

M. and make twenty pills. Sig. From five to eight pills per day.

---

**Menthol in Pruritis.**—According to the researches of Dubreuil and Archambault, who have studied the effects of menthol in a variety of pruritic affections, it is stated "that nothing can equal it for the relief of the itching of urticaria, itching eczema and pruritis ani, and all skin diseases where scratching keeps up the lesions of the skin."

In ordinary cases a ten per cent. solution in alcohol, olive or almond oil may be employed, or from two to five per cent. of menthol may be added to oxide of zinc ointment. Upon an excoriated surface or a mucous membrane, care must be exercised not to use too strong applications, as a disagreeable sensation of burning results in such cases.—*New Remedies.*

---

**Dr. Culver's Treatment of Carbuncle.**—About Nov., '89, my father, 86 years of age, complained for several days of sore back. On examination I found a carbuncle the size of my hand on the right side of the spinal column in the thoracic region. Poulticed it a few days, when about a dozen openings appearing, I concluded to try a 95 per cent. solution of carbolic acid, applied with a feather to the entire surface. He made no complaint, but next day said it was better. I made two more applications on the two succeeding days, and on the fourth day it appeaed well, no sloughing, no loss of tissue, and he had no further trouble with it. So quick and satisfactory a result surprised me, knowing the usual tedious character of carbuncle in this region.

I have since used it in several cases with uniform success and no untoward results, so that a disease which I used to regard as formidable, I no longer fear.—*Ex.*

**Phenacetine in Frequent Urination.**—Dr. Traill Geeen, in the *University Medical Magazine*, recommends phenacetine to be given to elderly patients whose rest at night is disturbed by the necessity of frequent rising for urination.

He prescribes ten grain doses at bedtime. Many old people, the majority of whom present excess of uric acid or urates in the urine, acquire the habit of too frequent urination. In many cases there may be irritability of the bladder. During the past year, Dr. G. attended a patient for whom he had prescribed for a year or two for frequency of passing urine. While under treat ment for another affection, he had occasion to prescribe a ten grain dose of phenacetine at bedtime, and learned the following morning that the patient had passed the night without a call to pass his water. The medicine was continued in ten grain doses for several nights, and rest of eight hours was secured. Since that time, the writer has verified his experience in the first case. The effect does not depend upon any property of the remedy to produce sleep, since the patient may wake without being called upon to urinate, and sulphonal and other remedies of the same class do not act in giving rest like phenacetine.—*Clinique.*

---

**Treatment of Gonorrhœal Vaginitis.**—The woman, if possible, should be kept in bed, and salol and an alkali administered internally for the relief of painful micturition. A lotion of bichloride solution 1:5,000 is used for bathing the outer parts and for injecting into the vagina three or four times a day for the first three days. After this, the strength is reduced 1:10,000. If this injection be painful carbolic acid solution is substituted for it. The injection should be taken with the patient in a recumbent posture. About the third day a speculum can be used and the cervix exposed to view. The parts may then be swabbed with strong carbolic acid or with a solution of bichloride of mercury, and this followed by dusting the parts with powdered iodoform. Later on a five per cent. solution of nitrate of silver may be painted over the cervix and vagina, and the vagina tamponed with iodoform gauze so as to separate its walls, the ends of the tampon being brought down to the vulva. If these are changed every two days the discharge will usually cease within a week or ten days. Before discharging the case as cured, never omit to examine the glands of Bartholini, and if any pus can be made to exude from them you may rest assured that the disease has not been eradicated. They should then be incised under cocaine anesthesia and touched with strong carbolic acid. If there be purulent discharge from the cervix and the Nabothian glands are distended, curette them and apply pure carbolic acid, using the iodoform tampon to prevent reinfection of the vagina.—*The American Journal of Obstetrics.*

---

**Treatment of Eczema in the French Hospitals.**—Dr. Jacquet (*La Semaine Medicale*) advises that attention be paid to the general health, which subject need not be considered here. In eczema in the folds of the skin, or where a parasitic origin is suspected, the writer begins with a calomel salve:

℞ Calomel . . . . . . . . . . . . . . grs. viij–xxij
Oxide of zinc . . . . . . . . . . . . . . . grs. xlv
Pure vaseline . . . . . . . . . . . . ʒ iv

After its application, dust on a powder of ten parts of the oxide of zinc and forty parts of talc.

If the disease be obstinate, then use the following ointment:

℞ Yellow oxide mercury . . . . . . . . grs. vij–xv

Oil of cade . . . . . . . . . . . . . . . ♏xv–ʒ j

Pure vaseline . . . . . . . . . . . . . . . . . ʒ v

If the eczema is on the hairy scalp, then use:

℞ Naphthol,
Camphor,
Resorcin . . . . . . . . . . . . . . āā grs. v–xv
Precipitated sulphur . . . . . . . . grs. xxx–ʒ jss
Pure vaseline . . . . . . . . . . . . . . . . ʒ v

Nitric acid solution (1.20) may be applied with a camel's hair brush, or a salve used containing pyrogallic or salicylic acid:

℞ Salicylic acid . . . . . . . . . . . . . . . . grs. vij
Pyrogallic acid . . . . . . . . . . . . . . . grs. xv
Pure vaseline . . . . . . . . . . . . . . . . . ʒ v

If these act as irritants, one may return to less energetic topical applications. The writer also uses plasters; the best of these contains the oxide of zinc. In rebellious cases one may employ the cod-liver oil as a local application. In pruriginous cases, one should use warm solutions of carbolic acid, together with a protective salve of the following consistence:

℞ Ethereal oil of peppermint or carbolic acid . ♏vij

Pure vaseline . . . . . . . . . . . . . . . ʒ ivss

Oxide of zinc . . . . . . . . . . . . . . . ʒ iij

Fissures are touched with a 15% solution of argentic nitrate.—*Lancet-Clinic.*

---

**For Neuralgia.**—Dr. Goss says take menthol, saturated tinct. 1 oz., chloroform 1 oz., tincture of camphor 1 oz,; apply over the pain. This also relieves headache of a nervous character.—*Ex.*

